# Vitamin A carotenoids, but not retinoids, mediate the impact of a healthy diet on gut microbial diversity

**DOI:** 10.1186/s12916-024-03543-4

**Published:** 2024-08-07

**Authors:** Ana M. Valdes, Panayiotis Louca, Alessia Visconti, Francesco Asnicar, Kate Bermingham, Ana Nogal, Kari Wong, Gregory A. Michelotti, Jonathan Wolf, Nicola Segata, Tim D. Spector, Sarah E. Berry, Mario Falchi, Cristina Menni

**Affiliations:** 1 Nottingham NIHR Biomedical Research Centre at the School of Medicine, University of Nottingham, Nottingham, NG5 1PB UK; 2 Inflammation, Recovery and Injury Sciences, School of Medicine, University of Nottingham, Nottingham, NG7 2UH UK; 3 Department of Twin Research & Genetic Epidemiology, King’s College London, London, SE1 7EH UK; 4 Human Nutrition and Exercise Research Centre, University of Newcastle, Newcastle Upon Tyne, NE2 4HH UK; 5 Centre for Biostatistics, Epidemiology, and Public Health, Department of Clinical and Biological Sciences, University of Turin, 10124 Turin, Italy; 6 Department CIBIO, University of Trento, Via Sommarive 9, 38123 Povo, Trento, Italy; 7 Department of Nutritional Sciences, King’s College London, 150 Stamford Street, London, SE1 9NH UK; 8 Zoe Limited, 164 Westminster Bridge Rd, London, SE1 7RW UK; 9 Department of Epidemiology, Harvard T.H. Chan School of Public Health, Boston, MA 02115 USA; 10 Metabolon Inc, Research Triangle Park, Morrisville, NC 27560 USA

**Keywords:** Vitamin A metabolites, Carotenoids, Retinoids, Gut microbiome composition

## Abstract

**Background:**

Vitamin A is essential for physiological processes like vision and immunity. Vitamin A’s effect on gut microbiome composition, which affects absorption and metabolism of other vitamins, is still unknown. Here we examined the relationship between gut metagenome composition and six vitamin A-related metabolites (two retinoid: -retinol, 4 oxoretinoic acid (oxoRA) and four carotenoid metabolites, including beta-cryptoxanthin and three carotene diols).

**Methods:**

We included 1053 individuals from the TwinsUK cohort with vitamin A-related metabolites measured in serum and faeces, diet history, and gut microbiome composition assessed by shotgun metagenome sequencing. Results were replicated in 327 women from the ZOE PREDICT-1 study.

**Results:**

Five vitamin A-related serum metabolites were positively correlated with microbiome alpha diversity (*r* = 0.15 to *r* = 0.20, *p* < 4 × 10^−6^). Carotenoid compounds were positively correlated with the short-chain fatty-acid-producing bacteria *Faecalibacterium prausnitzii* and *Coprococcus eutactus.* Retinol was not associated with any microbial species. We found that gut microbiome composition could predict circulating levels of carotenoids and oxoretinoic acid with AUCs ranging from 0.66 to 0.74 using random forest models, but not retinol (AUC = 0.52).

The healthy eating index (HEI) was strongly associated with gut microbiome diversity and with all carotenoid compounds, but not retinoids. We investigated the mediating role of carotenoid compounds on the effect of a healthy diet (HEI) on gut microbiome diversity, finding that carotenoids significantly mediated between 18 and 25% of the effect of HEI on gut microbiome alpha diversity.

**Conclusions:**

Our results show strong links between circulating carotene compounds and gut microbiome composition and potential links to a healthy diet pattern.

**Supplementary Information:**

The online version contains supplementary material available at 10.1186/s12916-024-03543-4.

## Background

Vitamins are micronutrients with antioxidant and neuroprotective properties. In addition to these functions, some vitamins have been linked to changes in the composition and diversity of the gut microbiome [1]. The human body requires 13 vitamins for proper physiological function, which can be divided into two categories, fat-soluble (vitamins A, D, E, and K), and water-soluble vitamins (8 B vitamins, and vitamin C).

Fat-soluble vitamins, particularly vitamins A, and D, have been studied for their effects on the immune system when absorbed in the gastrointestinal tract and secreted into the bloodstream [2]. A recent cross-sectional study of 567 older people found that those with higher levels of vitamin D had a greater abundance of butyrate-producing bacteria in the gut [3]. However, the relationship between vitamin A levels and the gut microbiome remains under-researched.

Vitamin A is an essential macronutrient for maintaining the integrity of the gut epithelial barrier [4, 5]. It supports the production and maintenance of mucus, tight junction proteins [6], and antimicrobial peptides, all of which protect the gut lining from pathogenic invasion and maintain a stable environment for the microbiota. A healthy gut barrier prevents dysbiosis (microbial imbalance) and supports a diverse microbial ecosystem [7]. Moreover, vitamin A has anti-inflammatory properties [8], which can help mitigate chronic inflammation in the gut [9]. By reducing inflammation and supporting the gut epithelial barrier, vitamin A fosters a more stable and diverse microbial environment increasing the abundance of beneficial commensals and decreasing the abundance of less favourable ones.

Vitamin A is derived from both retinyl acids found in animal-derived foods, or through a series of enzymatic reactions from carotenes and carotenoids in plant-based foods. Although the term vitamin A is mostly associated with retinol, and retinol is, in fact, the predominant form of retinoids in the human body, the main biologically active molecules are the oxidised derivates 11-*cis*-retinal and all-*trans*-retinoid acid (ATRA) [10]. ATRA acts as the active form, binding to retinoic acid receptors.

More than 80% of vitamin A in the liver is stored in hepatic stellate cells [11]. In hepatocytes, retinyl esters are hydrolysed by retinyl ester hydrolase to generate retinol, which subsequently binds to retinol-binding protein (RBP), before being released into circulation, where it is up-taken by systemic cells via membrane receptors, such as STRA6 [12]. The process of mobilising retinol is tightly regulated by variables that govern the rates of synthesis and secretion of RBP [10].

Since, the human body cannot produce vitamin A, it must be obtained from the diet, either as preformed vitamin A, found in foods of an animal origin, or as provitamin A carotenoids, found in several fruit and vegetables [10]. Milk and dairy products, as well as meat and its products, are the largest contributors of preformed vitamin A to the human diet, followed by eggs, egg products, and fish [10, 13].

Carotenoids can eventually be metabolised to retinol [14]. Retinoic acid has been shown to be an important determinant of intestinal immunity and permeability [15]. Importantly, absorption of dietary vitamin A depends on the fat-solubilising properties of bile acids [16], which are in turn modulated by gut microbiome composition, and bacterial conjugation of secondary bile acids [17].

Given that diets rich in carotenoids are likely to support a healthy gut microbiome [18] and the multiple beneficial effects of vitamin A on immune function and gut barrier integrity [18, 19], we hypothesised a positive relationship between alpha diversity and vitamin A.

The purpose of this study is to investigate whether there is a connection between the composition and alpha diversity of the gut microbiome and the presence of vitamin A-related metabolites, specifically retinoids and carotenoids, in the bloodstream and stool, using machine learning methods. We further quantify the extent to which bile acids influence the levels of retinoids and carotenoids. Finally, we investigate the role that vitamin A metabolites play in the favourable association between the healthy eating index (HEI) [20] and the composition of the gut microbiome.

## Methods

### Discovery cohort

Study participants were individuals enrolled in the TwinsUK registry, a national register of adult twins recruited as volunteers without selecting for any particular disease or trait [21]. Twins provided informed written consent, and the study was approved by St. Thomas’ Hospital Research Ethics Committee (REC Ref: EC04/015). Here we included 1053 individuals with concurrent vitamin A metabolites, bile acids measured by mass spectrometry, and gut microbiome profiled by shotgun metagenome. A subset of the included individuals also had available data on the quality of their habitual diet, as measured by the HEI [20].

### Replication cohort

The replication cohort consisted of an independent sample of 327 females from the UK-based ZOE PREDICT-1 study [22]—in a post hoc analysis, with serum and stool vitamin A metabolites measured by Metabolon Inc. as well as whole-shotgun metagenomic data available, and who completed a food frequency questionnaire (FFQ). Ethical approval for ZOE PREDICT-1 was obtained from St. Thomas Hospital research ethics committees. All individuals provided informed written consent (IRAS 236407) and the trial was registered on ClinicalTrials.gov (registration number: NCT03479866).

### Vitamin A metabolomics profiling

Vitamin A concentrations were measured from stool and serum samples by Metabolon Inc. (Durham, USA) using an untargeted LC–MS platform, as previously described [23, 24]. Six vitamin A-related metabolites were detected in serum, of which two retinols and four carotenoids, while five vitamin A-related metabolites were detected in stool (one retinol and four carotenoids). To remove batch variability from vitamin A profiling, for each vitamin A-related metabolite, the values in the experimental samples were divided by the median of those samples in each instrument batch, giving each batch and thus each vitamin A metabolite a median of one. Vitamin A metabolites with more than 80% missing measurements were excluded, and those with missingness between 20 and 80% were dichotomised. Vitamin A metabolites present in over 80% of the sample were batch normalised and inverse normalised. After cleaning, 6 vitamin A metabolites in serum (2 retinols and 4 carotenoids) and 5 in stool (1 retinol and 4 carotenoids) remained. Carotenoid metabolites include beta-cryptoxanthin, and carotene diol (1–3). Retinol metabolites include 4-oxoretinol acid (oxoRA) and retinol.

### Bile acid metabolomics profiling

Circulating primary bile acids, including cholic acid (CA), chenodeoxycholic acid (CDCA), taurocholic acid (TCA), glycocholic acid (GCA), taurochenodeoxycholic acid (TCDCA), and glycochenodeoxycholic acid (GCDCA) were measured from the same serum samples by Metabolon Inc. (Durham, USA) using an untargeted LC–MS platform, as described above.

### Dietary information

A validated 131-item semi-quantitative food frequency questionnaire (FFQ) established for the European Prospective Investigations into Cancer and Nutrition (EPIC)-Norfolk study [25] was used to estimate habitual dietary information. From FFQs, food item, macro- and micronutrient intakes were determined using FETA software [25, 26], we then calculated indexes to represent the whole dietary pattern, including HEI, which characterises intakes of foods and nutrients and is understood to be associated with various chronic diseases [27].

### Microbiome sequencing and profiling

Deep shotgun metagenomic sequencing in stool samples from TwinsUK and ZOE PREDICT-1 and its profiling was performed as previously described [28, 29]. To reduce noise caused by species with low prevalence and improve power to detect statistically significant species, we removed species with a prevalence < 20%.

### Statistical analysis

Statistical analyses were performed using Stata version 18 and R version 4.3.1 [30].

For alpha diversity, we constructed several metrics to quantify diversity of the gut microbiome within individual participants. The Shannon diversity index also called Shannon Entropy [31] uses Claude Shannon’s formula for entropy to estimate species diversity [22] and the Simpson diversity index, which gives more weight to common or dominant species, were calculated using the ‘diversity’ function in the ‘vegan’ R package [32]. Observed richness, which is simply the count of species observed, was calculated using the ‘specnumber’ function. To account for the twin nature of our data, linear mixed models adjusting for age, sex, body mass index (BMI) as fixed effect, and family structure, as random effect, were used independently in both TwinsUK and ZOE PREDICT-1 to investigate the univariate associations between vitamin A-related metabolites and gut microbiome characteristics. Specifically, we investigated associations with several alpha diversity metrics and at the taxonomic level with inverse normalised species abundances. *P*-values were corrected for multiple testing using Bonferroni correction, and associations with adjusted *P*-value < 0.05 were considered as significant. Results were combined using fixed effect meta-analyses. To assess between participant differences (beta diversity) and vitamin A-related metabolite levels, we constructed a Bray–Curtis dissimilarity matrix from the gut microbial community data, and we performed a PERMANOVA (1000 simulations) using the ‘adonis’ function (‘vegan’ package [32]) to determine significance, adjusting for age, sex, and BMI.

To quantify how much of the vitamin A-related metabolite levels could be predicted using gut metagenome data, we used random forest (RF) models from the randomForest package in R [33] (version 4.7–1.1). Models were conducted in TwinsUK and ZOE-PREDICT-1 data independently. We split the dataset into a training set (80%) and a test set (20%), which was held out to test performance. In the training data, predictors with zero or near-zero variance were excluded using the ‘nearZeroVar’ function (caret R package [34]). Hyperparameters, *mtry* (number of variables randomly sampled as candidates for each split) and *min.node.size* (minimum size of terminal nodes in each tree) were tuned using fivefold adaptive resampling (caret package in R [34]) with 3 repeats. The optimal number of features for each model was decided by fivefold recursive feature elimination (‘rfcv’ function from the randomForest package [33]). We iteratively removed features by assessing the model’s performance against the cross-validated error rate to determine the optimal subset of features (smallest number of features with smallest error rate). The model was retrained on the training data and the predictive performance tested using the test set. The performance of each model was assessed using the area under the receiver operating characteristic (AUC), which measures the model’s capacity to discriminate different classes and Spearman’s correlations between the model’s predicted class and the true class to quantify the model’s accuracy and reliability.

Linear mixed models were also employed to investigate the associations between vitamin A-related metabolites and (i) serum levels of bile acids and (ii) HEI, adjusting for age, sex, BMI, and family relatedness as random effects.

To investigate whether vitamin A-related metabolites mediate the relationship between a healthy diet and gut microbiome diversity, we conducted causal mediation analyses using the Baron and Kenny approach [35]. We first tested the three mediation assumptions and we then performed causal mediation analysis using the ‘mediate’ function in the R package ‘mediation’ (version 4.5.0) [36]. Each analysis was performed independently for each vitamin A metabolite and adjusted for age, sex, and BMI. A significant mediatory effect was determined by the significance (*p* < 0.05) and magnitude of the indirect effect. We further computed the variance accounted for (VAF) as the ratio of the indirect-to-total effect, which describes the proportion of the variance explained by the mediation process, e.g. the proportion of the effect of a healthy diet on gut microbiome that goes through the vitamin A metabolite. We used Vanderweele’s sensitivity analysis [37, 38] to evaluate the robustness of the estimated mediation effects to unmeasured confounding. This involves assessing the potential impact of unmeasured confounders on both the mediator-outcome and exposure-mediator relationships.

## Results

**Fig. 1:**
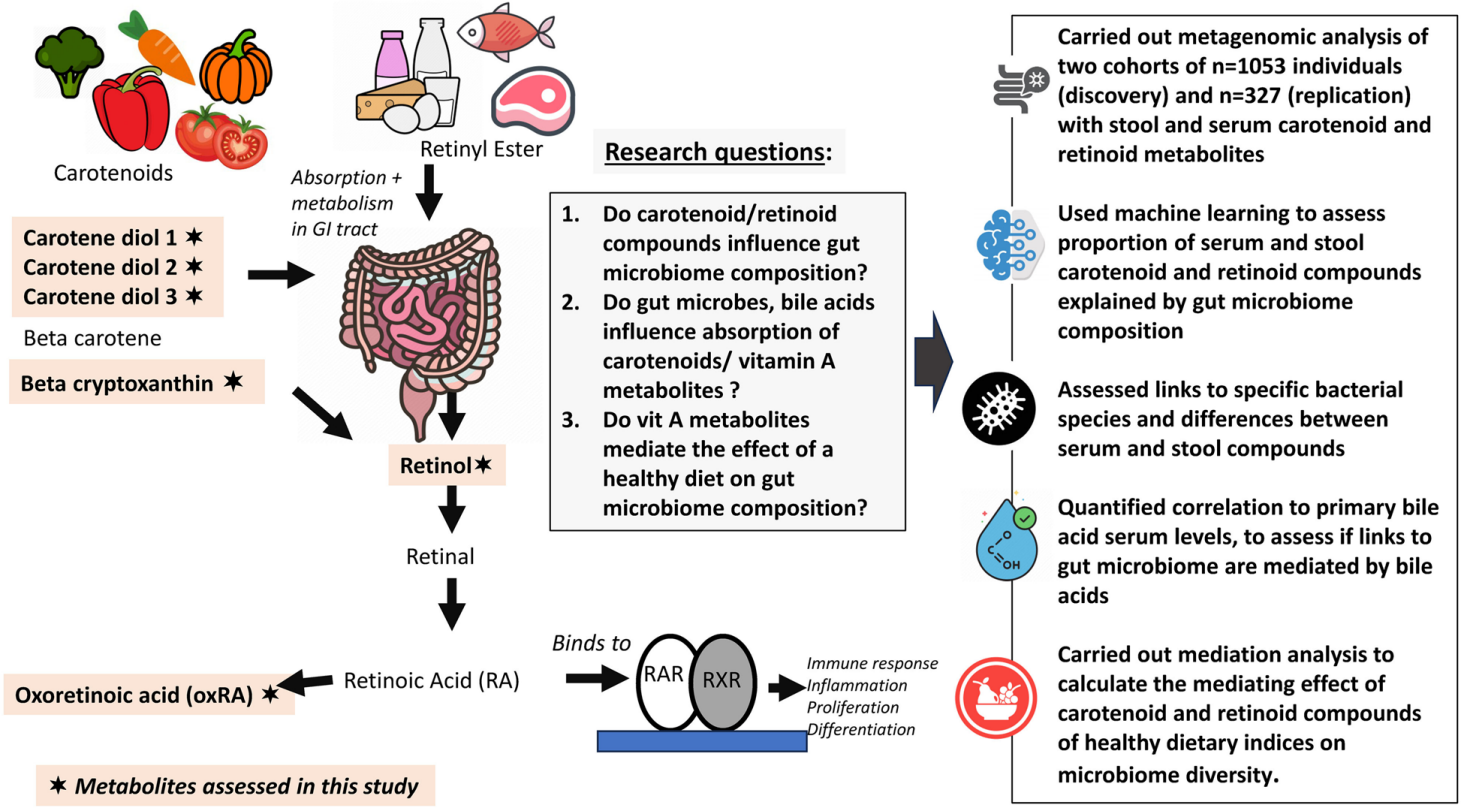
Schematic representation of the study. The figure describes the links between dietary intake and the studied vitamin A-related metabolites, and highlights the research questions we are addressing, the data, and the analytical workflow used

A flowchart of the study design is presented in Fig. 1.

The descriptive characteristics of the study populations are presented in Table 1. We included 1053 individuals from the TwinsUK cohort [21], and 327 female individuals from the ZOE PREDICT-1 study [21, 22], with vitamin A metabolites measured in serum (*n* = 6) and stool (*n* = 5) by Metabolon Inc. [24], and gut microbiome composition assessed by whole-shotgun metagenomics. The average (standard deviation, SD) age in TwinsUK was 57.72 (15.20), and in ZOE PREDICT-1 53.80 (7.00) years, while average body mass index (BMI) was 26.18 (5.15) and 26.24 (5.62) kg/m^2^, respectively.

**Table 1:** Descriptive characteristics of the study populations

Phenotype	TwinsUK	ZOE-PREDICT 1
*N*	1053	327
Females, *n* (%)	884 (83.95%)	327 (100%)
Age, years (SD)	57.72 (15.20)	53.80 (7.00)
BMI, kg/m^2^	26.18 (5.15)	26.24 (5.62)
HEI (SD)	59.27 (9.93)	65.42 (7.02)
Shannon diversity index (SD)	3.91 (0.54)	4.23 (0.38)

*BMI* body mass index, *HEI* healthy eating index

### Vitamin A metabolites and gut microbiome association

**Fig. 2:**
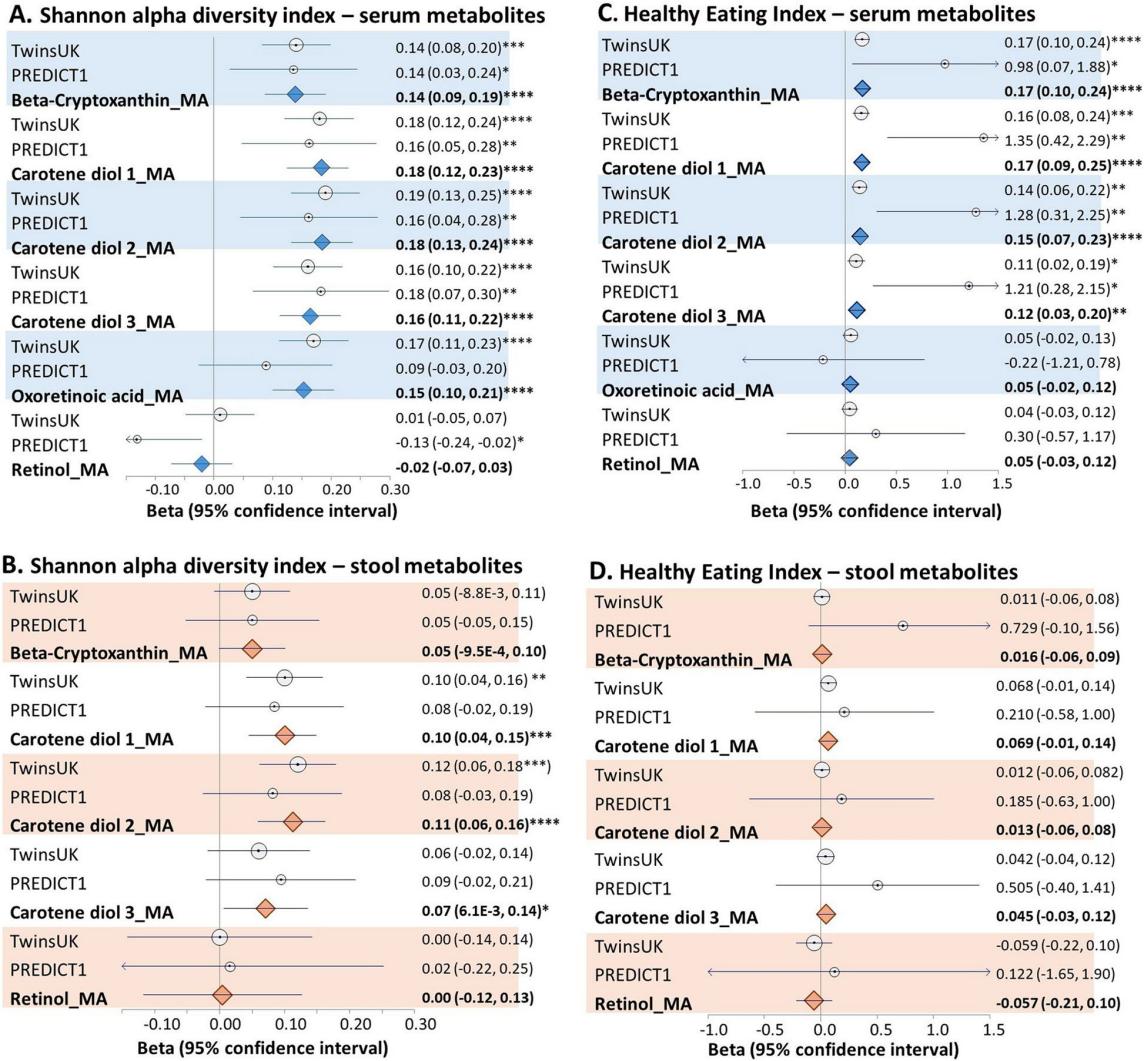
Associations between vitamin A metabolites from serum and stool and the Shannon diversity index (A and B) and the healthy eating index (C and D). Data shown. Circles show effect sizes in the TwinsUK and ZOE PREDICT 1 cohorts, diamonds are summary effect sizes derived from fixed effects meta-analysis (MA) combining both cohorts. Whiskers represent the 95% confidence intervals. Statistical significance is indicated by asterisks as follows: * *p* < 0.05; ** *p* < 0.01; *** *p* < 0.001; **** *p* < 0.0001

All vitamin A-related serum metabolites except retinol were positively correlated with the Shannon diversity index after adjusting for age, sex, BMI, and family relatedness in the TwinsUK cohort (Fig. 2, panels A and B). Further adjusting for history of cardiovascular diseases (including cerebrovascular disease, heart failure, ischemic heart disease, coronary artery disease, and atrial fibrillation), type 2 diabetes, chronic obstructive pulmonary disease, allergy, diet (including healthy eating index, fibre intake, vegetable intake, and energy intake), antibiotics use, physical exercise, vitamin supplementation, and sequencing depth, did not change the results (Additional File 1:TableS1). Consistent results were observed in ZOE-PREDICT 1 (Fig. 2, panels A and B) and when investigating the correlation with the Simpson index and with the number of observed species (Additional File 1:TableS2). Moreover, we detected a significant relationship between vitamin A metabolites and beta-diversity (as determined by Bray–Curtis indices), after adjusting for age, sex, and BMI (Additional File 1:TableS3).

**Fig. 3:**
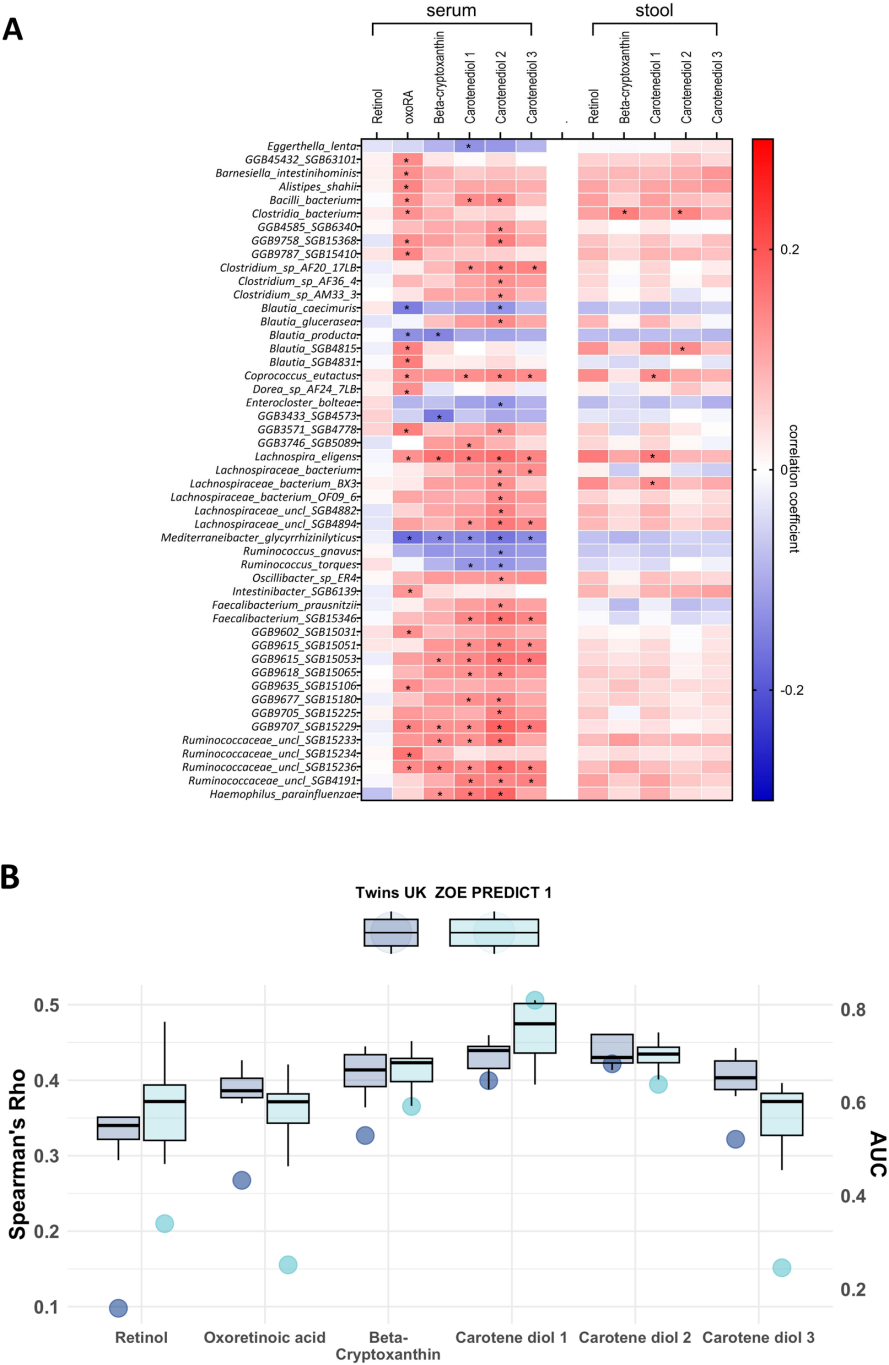
**A** Heatmap representing correlation of vitamin A-related metabolites from serum and gut bacterial species. Each cell of the matrix contains the regression coefficient between one vitamin A-related metabolite and a bacterial species. The table is colour-coded by correlation according to the table legend (red for positive and blue for negative correlations). * *p* < 0.05; ** *p* < 0.01; *** *p* < 0.001; **** *p* < 0.0001. **B **Prediction of the gut microbiota in vitamin A-related metabolites estimated by random forestregressors (using Spearman’s correlations) and classifiers (using AUC values) in TwinsUKand ZOE PREDICT-1 participants. Boxplots represent the mean AUC and the 95% confidence intervals across fivefold for TwinsUK and ZOE PREDICT-1. Dark blue and light blue circles indicate the mean of Spearman’s correlations between the real value of each vitamin A-related metabolite and the value predicted by the models across fivefold in TwinsUK and ZOE PREDICT-1

We then investigated, in TwinsUK, the univariate association between vitamin A-related metabolites and gut microbial species with a prevalence > 20%, identifying 96 significant associations (Bonferroni < 0.05), involving 49 unique bacterial species (Fig. 3A, Additional File 1:TableS4). Similar but weaker associations were seen with stool metabolite levels (Fig. 3A). Among the associated species, we identified *Faecalibacterium prausnitzii* and *Coprococcus eutactus* to be positively correlated with all carotenoid metabolites, whereas negative correlations included species such as *Ruminococcus torques* and *R. gnavus* and *Eggerthella lenta*,* Tyzzerella nexilis*, and *Flavonifractor plautii*. No association was found between individual taxa and serum or stool levels of retinol.

Next, we used random forest models to measure the ability of microbial species abundances to predict circulating vitamin A-related metabolite levels and generate a quantitative estimate of the extent to which gut microbiome composition is linked to vitamin A metabolite levels underscoring the importance of gut health in overall nutritional and anti-inflammatory status. The model performance was evaluated using the area under the receiver operating characteristic curve (AUC) and calculating Spearman’s correlations between predicted and observed values (Rho). In TwinsUK, on average, the gut microbiome was able to predict circulating levels of carotenoid metabolites across the 5-folds (Fig. 3B), with carotene diol (2) presenting the strongest association (AUC [95%CI] = 0.74 [0.67; 0.81], rho [95%CI] = 0.31 [0.15, 0.45]).

We performed a sensitivity analysis to explore whether our results were dependent on the transformation used on the gut microbiome (inverse normalisation). We transformed the data using the centred log-ratio (CLR) transformation and results were consistent (e.g. carotenediol (2) AUC [95% CI] = 0.68 [0.6; 0.76], rho [95% CI] = 0.25 [0.1; 0.4]).

We replicated the predictive models in the ZOEPREDICT-1 cohort. Consistent with the observations in TwinsUK, the gut microbiome was able to predict circulating levels of carotenoid metabolites with the largest AUC for carotene diol (2) (0.73 [0.60, 0.86]) (Fig. 3B).

### Vitamin A metabolites and bile acid levels

In TwinsUK, we quantified the effect of circulating primary bile acids on carotenoid and retinoid circulating levels to test whether fat absorption, which is modulated by primary BAs [4], influenced serum vitamin A levels. We tested their correlation with CA and CDCA, and their conjugated bile salts TCA, GCA, TCDCA, and GCDCA, which have been suggested in the literature to influence vitamin A absorption and metabolism [16], and which are known to have strong links to gut microbiome composition and function [39, 40]. We found three vitamin A-related metabolites nominally associated with bile acid levels: carotene diol (2) was negatively correlated with both GA (beta [95% CI] =  − 0.08 [− 0.14, − 0.07], *p* = 0.01) and GCDCA (beta [95% CI] =  − 0.06 [− 0.12, − 0.004], *p* = 0.03), carotene diol (1) was positively associate with CA (beta [95% CI] = 0.07[0.001,0.15], *p* = 0.05], while oxoRA was positively associated with CA (beta [95% CI] = 0.07, [0.001, 0.16], *p* = 0.045, and 0.12 [0.05, 0.12], *p* = 5.43 × 10^−4^, respectively, Additional File 1:Fig. 1). After adjusting for multiple testing (Bonferroni *p*-value = 0.05/30 = 1.6 × 10^−3^), only oxoRA was associated with CA levels. None of the carotenoids nor retinol were correlated with primary bile acids after adjustment for multiple testing.

### Vitamin A metabolites and diet quality association

We further investigated the association between serum and stool vitamin A-related metabolites, and the quality of habitual diet, as measured by the HEI in a subset of 664 TwinsUK individuals with FFQs available. We found strong positive associations between serum carotenoids and HEI after adjusting for covariates, while no effects were observed for retinols or for stool vitamin A metabolite abundances (Fig. 2C). Results were replicated in PREDICT 1 (Fig. 2D).

**Fig. 4:**
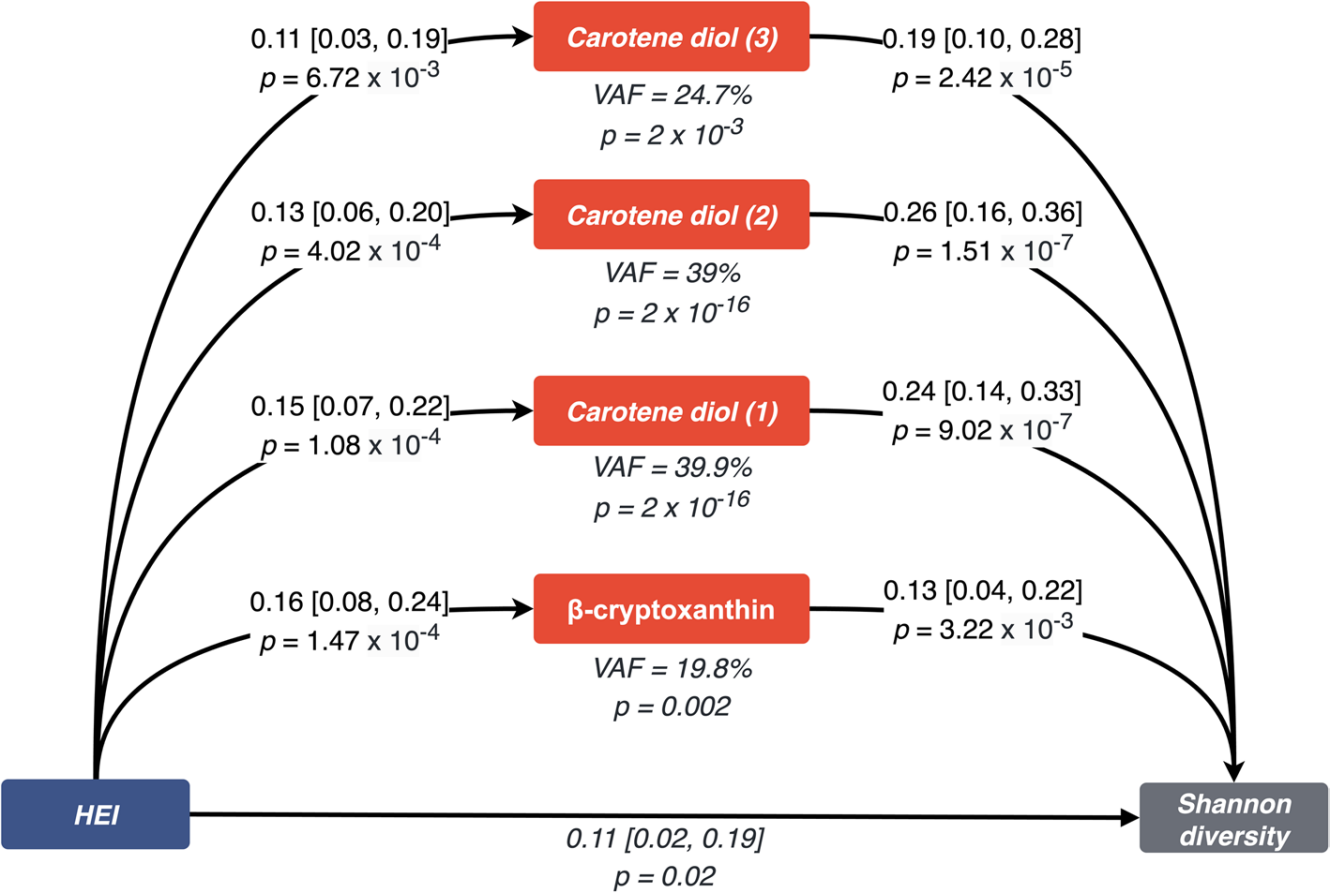
Mediation analysis diagram. A healthy diet, represented by the healthy eating index, was modelled as the exposure. Alpha diversity of the gut microbiome, measured by the Shannon diversity index, was modelled as the outcome. Each vitamin A metabolite was modelled as a mediator. Summary statistics from the mediation analysis are depicted as beta (95% confidence interval). Abbreviations: HEI, healthy eating index; CI, confidence interval

Consistent with the literature [41], we found a positive association between the HEI and the Shannon diversity index (beta [95% CI] = 0.10 [0.02, 0.19],* p* = 0.01) after adjusting for age, sex, BMI, and family relatedness. Importantly, when adjusting for each of the carotenoids in turn, the effects were attenuated (Additional File 1:TableS5). We, therefore, performed a formal mediation analysis adjusting for age, BMI, and sex to determine whether the serum carotenoid metabolites mediate the association between HEI and the Shannon diversity index. We only focused on carotenoids mediating the effects of healthy diet on the microbiome and not vice versa as experimental evidence has shown that in anaerobic conditions (as is the case in the human colon) that gut microbes cannot produce carotenoids [42, 43]). All four carotenoid metabolites met the criteria for a mediator, as laid out by Baron and Kenny [35]. Following a formal mediation analysis, all four carotenoids significantly mediated the effect of a healthy diet (HEI) on gut microbial diversity (Fig. 4). The variance accounted for ranged from 19.8% for beta-cryptoxanthin to 39.9% for carotene diol (1).

However, our findings surrounding carotene diol (1) and (2) could be sensitive to unmeasured residual confounding (*e*-values = 1.32 [LowerBound = 1.0] [37, 38] (Additional File 1:TableS6). On the other hand, for carotene diol (3) and beta-cryptoxanthin, our results appear robust (*e*-values = 1.35 [LowerBound = 1.04].

## Discussion

In this large-scale study investigating the links between stool and serum vitamin A-related metabolites and the gut microbiome, we report that carotenoids are strongly correlated with gut microbiome composition, while retinoids are only weakly associated with gut microbes.

In addition to strong positive associations with alpha diversity, we also observed significant positive correlations between circulating levels of carotenoid compounds and the abundance of short-chain fatty acid (SCFA) producing gut microbes, such as *Faecalibacterium prausnitzii* and *Coprococcus eutactus* [44–46]. Carotenoids are known for their antioxidant properties [47], which may contribute to a higher abundance of beneficial bacterial species. Similarly, we found that carotenoids were negatively correlated with species previously linked to inflammation (*Ruminococcus torques*, *Ruminococcus gnavus*) [48], or unfavourable cardiometabolic outcomes [29] as well as colorectal cancer risk and progression (*Eggerthella lenta*,* Tyzzerella nexilis*,* Flavonifractor plautii*) [49–51].

Consistently, the levels of these compounds in serum, though much less in stool, were partially predicted by the gut microbiome composition using random forest models and, importantly, these results were replicated in an independent cohort. The lack of prediction of the gut microbiome on stool carotenoid and retinoid levels may be reflecting that absorption of these substances takes place mostly in the proximal intestine (jejunum and duodenum) [52] and hence it is likely that the systemic effects of carotenoids and retinoids influence the gut microbiome and not gut microbes affecting absorption of these fat-soluble molecules.

We also report that, although all these compounds are fat soluble, levels of primary bile acids (which are key in the absorption of fat substances and have been implicated in the absorption and metabolism of vitamin A and its metabolites [16]) are not correlated with circulating levels of carotenoid compounds or of retinol. Strong links between bile acid metabolism and gut microbiome composition have been reported [17, 40], but the associations reported here with gut microbiome diversity are unlikely to be caused by the effects of the gut microbiome on absorption in the upper GI tract, suggesting that this mechanism may play only a small role in determining levels of circulating carotenoids and retinoids within the ranges seen in a healthy population. Indeed, recent experimental data has shown the inability of gut microbes to synthesise carotenoids in anaerobic conditions comparable to the human gut [43], hence our data are consistent with dietary carotenoids influencing the gut microbiome composition and not vice versa.

Finally, we showed that, to some extent, the effect of eating a healthy diet (as measured by adherence to HEI) on gut microbiome alpha diversity is mediated by levels of carotenoid compounds. The proportion of the effect ranged from 19.8% for beta-cryptoxanthin to 39.9% for carotene diol (1). No significant effect was seen for retinoid compounds. These results could have potential implications for personalised nutrition, and the development of dietary interventions to promote a healthy gut microbiome [53]. Indeed, these data highlight strong links between fat-soluble carotenoids and beneficial gut microbes, suggesting the potential of modulating some of these species by carotenoid intake. Further research in this area may contribute to the development of targeted dietary recommendations for individuals aiming to optimise their gut microbial composition and promote overall health.

Our study has many strengths. First, we used a well-validated large population-based cohort for discovery and validated our findings in an independent cohort. Both cohorts had the same *omics* profiling and were processed using the same quality control and analysis pipelines. Second, we used whole-shotgun metagenomics data, which provides considerably more depth than characterisation via 16S rRNA gene amplicon data. Third, we measured vitamin A metabolites as well as several primary and secondary bile acids in both serum and stool using one of the best-characterised targeted commercially available metabolomic panels.

We also note some limitations. First, our study sample is predominantly female, and our replication cohort included females only. Second, vitamin A metabolite levels the metabolomics panel used provides relative values, and not absolute concentrations of the metabolites in serum or stool. This limitation also affects the clinical interpretability of our results, where absolute quantification is necessary for comparing results to clinical thresholds used to diagnose/manage vitamin A deficiency or toxicity. Nevertheless, our results indicate relative changes and associations facilitating deeper exploration within future studies and subsequent improved clinical relevance. Third, dietary intake was measured using a food frequency questionnaire, which has inherent biases, including response bias and potential misclassification. Fourth, our mediation analysis may not truly identify a causal relationship, due to a lack of a temporal relationship between measurement of our exposure, mediator, and outcome, and we are unable to exclude the possibility of residual unmeasured confounders, which highlights the need for further research into the interactions between vitamin A-related metabolites and the gut microbiome, and to understand what, if any, such unmeasured confounders might be. Nevertheless, we successfully replicated the results in both TwinsUK and ZOE Predict-1. Finally, we are unable to infer causality between carotenoids levels, gut microbiome composition, and HEI.

## Conclusions

In summary, this is, to the best of our knowledge, the first large-scale study investigating the links between vitamin A metabolites and the gut microbiome using whole-genome shotgun metagenomic sequencing. Here, we show that carotenoids but not retinoids are strongly correlated with higher alpha diversity and with higher relative abundance of beneficial bacteria, and that vitamin A metabolites mediate between 20 and 39% of the effects of adherence to a healthy diet on microbiome diversity.

### Supplementary Information

Supplementary Material 1: Table S1. Association between Shannon Diversity and vitamin A related metabolites adjusting for age, sex, BMI, history of cardiovascular diseases (i.e. cerebrovascular disease, heart failure, ischemic heart disease, coronary artery disease and atrial fibrillation), type 2 diabetes, chronic obstructive pulmonary disease, allergy, diet (i.e. healthy eating index, fibre intake, vegetable intake and energy intake), antibiotics use, physical exercise, vitamin supplementation, sequencing depth and family relatedness in TwinsUK. In this analysis, missing dietary values were imputed to the mean. Table S2. Association between Alpha Diversity indices and vitamin A related metabolites adjusting for age, sex, BMI and family relatedness in TwinsUK and PREDICT-1. Beta (SE) and P value for fixed effect meta-analysis are reported. Table S3. Beta-diversity estimates for gut microbiome composition in relation to serum and stool vitamin A-related metabolites using PERMANOVA (Bray-Curtis dissimilarity). Results of the PERMANOVA analysis as implemented in ADONIS, examining the association between gut microbiome beta-diversity using Bray-Curtis dissimilarity and serum and stool vitamin A-related metabolites. The table includes the R-squared (R²) values, and p-values for each metabolite unadjusted and adjusted for covariates (age, sex, BMI and family relatedness). The R² value represents the proportion of total variation in gut microbiome composition explained by each metabolite. P-values are derived from 1000 permutations and indicate the statistical significance of the observed associations. Table S4. Association between circulating vitamin A-related metabolites and bacterial species adjusting for age, sex, BMI, family relatedness and multiple testing in TwinsUK. Table S5. Association between Shannon and HEI, adjusted for age, sex, BMI, and family relatedness and the below carotenoids. Table S6. Sensitivity Analysis for Unmeasured Confounding using the E-value method. Figure S1. Association between vitamin A related metabolites and circulating primary bile acids.

## Data Availability

The data used in this study are held by the Department of Twin Research at King’s College London. The data can be released to bona fide researchers using our normal procedures overseen by the Wellcome Trust and its guidelines as part of our core funding (https://twinsuk.ac.uk/resources-for-researchers/access-our-data/). The gut microbiome data is available on EBI (https://www.ebi.ac.uk/) under accession number PRJEB39223 (ZOE- PREDICT-1) and PRJEB32731 (TwinsUK).

## Abbreviations

ATRA: All-*trans*-retinoid acid
BMI: Body mass index
CA: Cholic acid
CDCA: Chenodeoxycholic acid
FFQ: Food frequency questionnaire
GCA: Glycocholic acid
GCDCA: Glycochenodeoxycholic acid
HEI: Healthy eating index
oxoRA: 4-Oxoretinol acid
RAR: Retinoic acid receptors
RBP: Retinol-binding protein
TCA: Taurocholic acid
TCDCA: Taurochenodeoxycholic acid

## References

[1] Rinninella E, Mele MC, Merendino N, Cintoni M, Anselmi G, Caporossi A, et al. The Role of Diet, Micronutrients and the Gut Microbiota in Age-Related Macular Degeneration: New Perspectives from the Gut−Retina Axis. Nutrients. 2018;10:1677. 10.3390/nu10111677 PMC6267253 30400586

[2] Mora JR, Iwata M, von Andrian UH. Vitamin effects on the immune system: vitamins A and D take centre stage. Nat Rev Immunol. 2008;8:685–98. 19172691 10.1038/nri2378 PMC2906676

[3] Thomas RL, Jiang L, Adams JS, Xu ZZ, Shen J, Janssen S, et al. Vitamin D metabolites and the gut microbiome in older men. Nat Commun. 2020;11:5997. 33244003 10.1038/s41467-020-19793-8 PMC7693238

[4] Vítek L, Haluzík M. The role of bile acids in metabolic regulation. J Endocrinol. 2016;228:R85-96. 26733603 10.1530/JOE-15-0469

[5] Cantorna MT, Snyder L, Arora J. Vitamin A and vitamin D regulate the microbial complexity, barrier function, and the mucosal immune responses to ensure intestinal homeostasis. Crit Rev Biochem Mol Biol. 2019;54:184–92. 31084433 10.1080/10409238.2019.1611734 PMC6629036

[6] Lima AAM, Soares AM, Lima NL, Mota RMS, Maciel BLL, Kvalsund MP, et al. Effects of vitamin A supplementation on intestinal barrier function, growth, total parasitic, and specific Giardia spp infections in Brazilian children: a prospective randomized, double-blind, placebo-controlled trial. J Pediatr Gastroenterol Nutr. 2010;50:309–15. 20038852 10.1097/MPG.0b013e3181a96489 PMC2830290

[7] Stolfi C, Maresca C, Monteleone G, Laudisi F. Implication of intestinal barrier dysfunction in gut dysbiosis and diseases. Biomedicines. 2022;10:10. 10.3390/biomedicines10020289 PMC8869546 35203499

[8] Reifen R. Vitamin A as an anti-inflammatory agent. Proc Nutr Soc. 2002;61:397–400. 12230799 10.1079/PNS2002172

[9] Kim D, Zeng MY, Núñez G. The interplay between host immune cells and gut microbiota in chronic inflammatory diseases. Exp Mol Med. 2017;49: e339. 28546562 10.1038/emm.2017.24 PMC5454439

[10] Carazo A, Macáková K, Matoušová K, Krčmová LK, Protti M, Mladěnka P. Vitamin A update: forms, sources, kinetics, detection, function, deficiency, therapeutic use and toxicity. Nutrients. 2021;13:1703. 34069881 10.3390/nu13051703 PMC8157347

[11] Blaner WS, Li Y, Brun P-J, Yuen JJ, Lee S-A, Clugston RD. Vitamin A absorption, storage and mobilization. Subcell Biochem. 2016;81:95–125. 27830502 10.1007/978-94-024-0945-1_4

[12] Bawa FNC, Zhang Y. Retinoic acid signaling in fatty liver disease. Liver Res. 2023;7:189–95. 37854944 10.1016/j.livres.2023.07.002 PMC10583737

[13] Al Binali HAH. Night blindness and ancient remedy. Heart Views. 2014;15:136–9. 25774260 10.4103/1995-705X.151098 PMC4348990

[14] Bonet ML, Canas JA, Ribot J, Palou A. Carotenoids and their conversion products in the control of adipocyte function, adiposity and obesity. Arch Biochem Biophys. 2015;572:112–25. 25721497 10.1016/j.abb.2015.02.022

[15] Bakdash G, Vogelpoel LTC, van Capel TMM, Kapsenberg ML, de Jong EC. Retinoic acid primes human dendritic cells to induce gut-homing, IL-10-producing regulatory T cells. Mucosal Immunol. 2015;8:265–78. 25027601 10.1038/mi.2014.64

[16] Saeed A, Hoekstra M, Hoeke MO, Heegsma J, Faber KN. The interrelationship between bile acid and vitamin A homeostasis. Biochim Biophys Acta Mol Cell Biol Lipids. 2017;1862:496–512. 28111285 10.1016/j.bbalip.2017.01.007

[17] Staley C, Weingarden AR, Khoruts A, Sadowsky MJ. Interaction of gut microbiota with bile acid metabolism and its influence on disease states. Appl Microbiol Biotechnol. 2017;101:47–64. 27888332 10.1007/s00253-016-8006-6 PMC5203956

[18] Eroglu A, Al'Abri IS, Kopec RE, Crook N, Bohn T: Carotenoids and Their Health Benefits as Derived via Their Interactions with Gut Microbiota. Adv Nutr. 2023;14(2):238-55. 10.1016/j.advnut.2022.10.007 PMC10229386 36775788

[19] McCullough FS, Northrop-Clewes CA, Thurnham DI. The effect of vitamin A on epithelial integrity. Proc Nutr Soc. 1999;58:289–93. 10466169 10.1017/S0029665199000403

[20] Guenther PM, Casavale KO, Reedy J, Kirkpatrick SI, Hiza HAB, Kuczynski KJ, et al. Update of the healthy eating index: HEI-2010. J Acad Nutr Diet. 2013;113:569–80. 23415502 10.1016/j.jand.2012.12.016 PMC3810369

[21] Verdi S, Abbasian G, Bowyer RCE, Lachance G, Yarand D, Christofidou P, et al. TwinsUK: the UK adult twin registry update. Twin Res Hum Genet. 2019;22:523–9. 31526404 10.1017/thg.2019.65

[22] Berry SE, Valdes AM, Drew DA, Asnicar F, Mazidi M, Wolf J, et al. Human postprandial responses to food and potential for precision nutrition. Nat Med. 2020;26:964–73. 32528151 10.1038/s41591-020-0934-0 PMC8265154

[23] Nogal A, Tettamanzi F, Dong Q, Louca P, Visconti A, Christiansen C, et al. A fecal metabolite signature of impaired fasting glucose: results from two independent population-based cohorts. Diabetes. 2023;72:1870–80. 37699401 10.2337/db23-0170 PMC10658071

[24] Attaye I, Beynon-Cobb B, Louca P, Nogal A, Visconti A, Tettamanzi F, Wong K, Michellotti G, Spector TD, Falchi M et al: Cross-sectional analyses of metabolites across biological samples mediating dietary acid load and chronic kidney disease. iScience. 2024;27(3):109132. 10.1016/j.isci.2024.109132 PMC10907771 38433906

[25] Bingham SA, Welch AA, McTaggart A, Mulligan AA, Runswick SA, Luben R, et al. Nutritional methods in the European prospective investigation of cancer in Norfolk. Public Health Nutr. 2001;4:847–58. 11415493 10.1079/PHN2000102

[26] Mulligan AA, Luben RN, Bhaniani A, Parry-Smith DJ, O’Connor L, Khawaja AP, et al. A new tool for converting food frequency questionnaire data into nutrient and food group values: FETA research methods and availability. BMJ Open. 2014;4: e004503. 24674997 10.1136/bmjopen-2013-004503 PMC3975761

[27] Kennedy ET, Ohls J, Carlson S, Fleming K. The healthy eating index: design and applications. J Am Diet Assoc. 1995;95:1103–8. 7560680 10.1016/S0002-8223(95)00300-2

[28] Visconti A, Le Roy CI, Rosa F, Rossi N, Martin TC, Mohney RP, et al. Interplay between the human gut microbiome and host metabolism. Nat Commun. 2019;10:4505. 10.1038/s41467-019-12476-z. 31582752 10.1038/s41467-019-12476-z PMC6776654

[29] Asnicar F, Berry SE, Valdes AM, Nguyen LH, Piccinno G, Drew DA, et al. Microbiome connections with host metabolism and habitual diet from 1,098 deeply phenotyped individuals. Nat Med. 2021;27:321–32. 10.1038/s41591-020-01183-8. 33432175 10.1038/s41591-020-01183-8 PMC8353542

[30] The R project for statistical computing. https://www.R-project.org. Accessed 30 Jan 2024.

[31] Konopiński MK. Shannon diversity index: a call to replace the original Shannon’s formula with unbiased estimator in the population genetics studies. PeerJ. 2020;8: e9391. 32655992 10.7717/peerj.9391 PMC7331625

[32] Community Ecology Package [R package vegan version 2.6–6.1]. 2024.

[33] Liaw A. Classification and regression by randomForest. R news. 2002;2(3):18–22.

[34] Kuhn M. Building predictive models in R using the caret package. J stat softw. 2008;28:1–26.27774042 10.18637/jss.v028.i05

[35] Baron RM, Kenny DA. The moderator-mediator variable distinction in social psychological research: conceptual, strategic, and statistical considerations. J Pers Soc Psychol. 1986;51:1173–82. 3806354 10.1037/0022-3514.51.6.1173

[36] Tingley D, Yamamoto T, Hirose K, Keele L, Imai K. mediation: R package for causal mediation analysis. J Stat Softw. 2014;59:1–38.26917999 10.18637/jss.v059.i05

[37] Smith LH, VanderWeele TJ. Mediational E-values: approximate sensitivity analysis for unmeasured mediator-outcome confounding. Epidemiology. 2019;30:835–7. 31348008 10.1097/EDE.0000000000001064 PMC6768718

[38] VanderWeele TJ. Bias formulas for sensitivity analysis for direct and indirect effects. Epidemiology. 2010;21:540–51. 20479643 10.1097/EDE.0b013e3181df191c PMC4231822

[39] Kiriyama Y, Nochi H. Physiological role of bile acids modified by the gut microbiome. Microorganisms. 2021;10:10. 35056517 10.3390/microorganisms10010068 PMC8777643

[40] Louca P, Meijnikman AS, Nogal A, Asnicar F, Attaye I, Vijay A, Kouraki A, Visconti A, Wong K, Berry SE et al: The secondary bile acid isoursodeoxycholate correlates with post-prandial lipemia, inflammation, and appetite and changes post-bariatric surgery. Cell Rep Med. 2023;4(4):100993. 10.1016/j.xcrm.2023.100993 PMC10140478 37023745

[41] Bowyer RCE, Jackson MA, Pallister T, Skinner J, Spector TD, Welch AA, et al. Use of dietary indices to control for diet in human gut microbiota studies. Microbiome. 2018;6:77. 29695307 10.1186/s40168-018-0455-y PMC5918560

[42] Lin D, Medeiros DM. The microbiome as a major function of the gastrointestinal tract and its implication in micronutrient metabolism and chronic diseases. Nutr Res. 2023;112:30–45. 36965327 10.1016/j.nutres.2023.02.007

[43] Matsumoto W, Takemura M, Nanaura H, Ami Y, Maoka T, Shindo K, et al. Carotenoid productivity in human intestinal bacteria Eubacterium limosum and Leuconostoc mesenteroides with functional analysis of their carotenoid biosynthesis genes. Engineering Microbiology. 2024;4: 100147. 10.1016/j.engmic.2024.100147 PMC11611032 39629323

[44] Martín R, Rios-Covian D, Huillet E, Auger S, Khazaal S, Bermúdez-Humarán LG, et al. Faecalibacterium: a bacterial genus with promising human health applications. FEMS Microbiol Rev. 2023;47(4):fuad039. 37451743 10.1093/femsre/fuad039 PMC10410495

[45] Nogal A, Louca P, Zhang X, Wells PM, Steves CJ, Spector TD, et al. Circulating levels of the short-chain fatty acid acetate mediate the effect of the gut microbiome on visceral fat. Front Microbiol. 2021;12: 711359. 34335546 10.3389/fmicb.2021.711359 PMC8320334

[46] Machiels K, Joossens M, Sabino J, De Preter V, Arijs I, Eeckhaut V, et al. A decrease of the butyrate-producing species Roseburia hominis and Faecalibacterium prausnitzii defines dysbiosis in patients with ulcerative colitis. Gut. 2014;63:1275–83. 24021287 10.1136/gutjnl-2013-304833

[47] Young AJ, Lowe GL. Carotenoids-antioxidant properties. Antioxidants (Basel). 2018;7:7. 29439455 10.3390/antiox7020028 PMC5836018

[48] Hall AB, Yassour M, Sauk J, Garner A, Jiang X, Arthur T, et al. A novel Ruminococcus gnavus clade enriched in inflammatory bowel disease patients. Genome Med. 2017;9:103. 29183332 10.1186/s13073-017-0490-5 PMC5704459

[49] Bai X, Wei H, Liu W, Coker OO, Gou H, Liu C, et al. Cigarette smoke promotes colorectal cancer through modulation of gut microbiota and related metabolites. Gut. 2022;71:2439–50. 35387878 10.1136/gutjnl-2021-325021 PMC9664112

[50] Lozano CP, Wilkens LR, Shvetsov YB, Maskarinec G, Park S-Y, Shepherd JA, et al. Associations of the dietary inflammatory index with total adiposity and ectopic fat through the gut microbiota, LPS, and C-reactive protein in the multiethnic cohort-adiposity phenotype study. Am J Clin Nutr. 2022;115:1344–56. 34871345 10.1093/ajcn/nqab398 PMC9071464

[51] Blacher E, Bashiardes S, Shapiro H, Rothschild D, Mor U, Dori-Bachash M, et al. Potential roles of gut microbiome and metabolites in modulating ALS in mice. Nature. 2019;572:474–80. 31330533 10.1038/s41586-019-1443-5

[52] Goncalves A, Roi S, Nowicki M, Dhaussy A, Huertas A, Amiot M-J, et al. Fat-soluble vitamin intestinal absorption: absorption sites in the intestine and interactions for absorption. Food Chem. 2015;172:155–60. 25442537 10.1016/j.foodchem.2014.09.021

[53] Ghosh TS, Valdes AM. Evidence for clinical interventions targeting the gut microbiome in cardiometabolic disease. BMJ. 2023;383: e075180. 37813434 10.1136/bmj-2023-075180 PMC10561016

