# Supplementary material for: Vitamin A carotenoids, but not retinoids, mediate the impact of a healthy diet on gut microbial diversity
Source: BMC Med. 2024 Aug 7;22:321. doi: 10.1186/s12916-024-03543-4 (PMC11304618; doi:10.1186/s12916-024-03543-4)

# **Table S1**. Association between Shannon Diversity and vitamin A related metabolites adjusting for age, sex, BMI, history of cardiovascular diseases (i.e. cerebrovascular disease, heart failure, ischemic heart disease, coronary artery disease and atrial fibrillation), type 2 diabetes, chronic obstructive pulmonary disease, allergy, diet (i.e. healthy eating index, fibre intake, vegetable intake and energy intake), antibiotics use, physical exercise, vitamin supplementation, sequencing depth and family relatedness in TwinsUK. In this analysis, missing dietary values were imputed to the mean.

| Tissue | Vitamin A | Beta | SE | P |
| --- | --- | --- | --- | --- |
| Serum | Retinol | -0.03 | 0.03 | 3.68E-01 |
| Serum | Oxoretinoic | 0.19 | 0.03 | 9.56E-12 |
| Serum | Beta-cryptoxanthin | 0.13 | 0.03 | 1.10E-05 |
| Serum | Carotenediol1 | 0.17 | 0.03 | 1.18E-07 |
| Serum | Carotenediol2 | 0.18 | 0.03 | 9.95E-09 |
| Serum | Carotenediol3 | 0.11 | 0.03 | 6.38E-04 |
| Stool | Retinol | 0.01 | 0.06 | 9.08E-01 |
| Stool | Beta-cryptoxanthin | 0.05 | 0.03 | 5.42E-02 |
| Stool | Carotenediol1 | 0.10 | 0.03 | 4.10E-04 |
| Stool | Carotenediol2 | 0.08 | 0.03 | 2.35E-03 |
| Stool | Carotenediol3 | 0.04 | 0.03 | 1.84E-01 |

# **Table S2.** Association between Alpha Diversity indices and vitamin A related metabolites adjusting for age, sex, BMI and family relatedness in TwinsUK and PREDICT-1. Beta(SE) and P value for fixed effect meta-analysis are reported.

| Tissue | Vitamin A metabolite | Alpha Diversity | Beta | SE | P |
| --- | --- | --- | --- | --- | --- |
| Serum | Retinol | Simpson | 0.00 | 0.03 | 8.98E-01 |
| Serum | Retinol | Observed Species | 0.02 | 0.03 | 5.12E-01 |
| Serum | Oxoretinoic | Simpson | 0.07 | 0.03 | 1.20E-02 |
| Serum | Oxoretinoic | Observed Species | 0.18 | 0.03 | 2.42E-11 |
| Serum | Bcryptoxanthin | Simpson | 0.07 | 0.03 | 9.48E-03 |
| Serum | Bcryptoxanthin | Observed Species | 0.11 | 0.03 | 1.04E-04 |
| Serum | Carotenediol1 | Simpson | 0.11 | 0.03 | 1.02E-04 |
| Serum | Carotenediol1 | Observed Species | 0.11 | 0.03 | 2.36E-04 |
| Serum | Carotenediol2 | Simpson | 0.11 | 0.03 | 8.95E-05 |
| Serum | Carotenediol2 | Observed Species | 0.12 | 0.03 | 2.01E-05 |
| Serum | Carotenediol3 | Simpson | 0.11 | 0.03 | 2.80E-04 |
| Serum | Carotenediol3 | Observed Species | 0.10 | 0.03 | 8.80E-04 |
| Stool | Retinol | Simpson | -0.01 | 0.06 | 9.31E-01 |
| Stool | Retinol | Observed Species | 0.05 | 0.06 | 4.04E-01 |
| Stool | Beta-cryptoxanthin | Simpson | 0.02 | 0.03 | 4.47E-01 |
| Stool | Beta-cryptoxanthin | Observed Species | 0.14 | 0.03 | 1.63E-07 |
| Stool | Carotenediol1 | Simpson | 0.06 | 0.03 | 2.98E-02 |
| Stool | Carotenediol1 | Observed Species | 0.14 | 0.03 | 1.79E-07 |
| Stool | Carotenediol2 | Simpson | 0.04 | 0.03 | 1.34E-01 |
| Stool | Carotenediol2 | Observed Species | 0.15 | 0.03 | 1.91E-08 |
| Stool | Carotenediol3 | Simpson | 0.01 | 0.03 | 8.11E-01 |
| Stool | Carotenediol3 | Observed Species | 0.14 | 0.03 | 2.24E-06 |

# **Table S3.** Beta-diversity estimates for gut microbiome composition in relation to serum and stool vitamin A related metabolites using PERMANOVA (Bray-Curtis dissimilarity). Results of the PERMANOVA analysis as implemented in ADONIS, examining the association between gut microbiome beta-diversity using Bray-Curtis dissimilarity and serum and stool vitamin A related metabolites. The table includes the R-squared (R²) values, and p-values for each metabolite unadjusted and adjusted for covariates (age, sex, BMI and family relatedness). The R² value represents the proportion of total variation in gut microbiome composition explained by each metabolite. P-values are derived from 1000 permutations and indicate the statistical significance of the observed associations.

| Matrix | Study cohort | metabolite | R² unadj | P-value | R² adj | P-value |
| --- | --- | --- | --- | --- | --- | --- |
| serum | TwinsUK | retinol | 0.0013 | 0.054 | 0.0012 | 0.090 |
|  |  | Oxoretinoic acid | 0.0047 | <0.001 | 0.0037 | <0.001 |
|  |  | Beta-cryptoxanthin | 0.0045 | <0.001 | 0.0039 | <0.001 |
|  |  | Carotene diol 1 | 0.0059 | <0.001 | 0.0054 | <0.001 |
|  |  | Carotene diol 2 | 0.0068 | <0.001 | 0.0064 | <0.001 |
|  |  | Carotene diol 3 | 0.0048 | <0.001 | 0.0046 | <0.001 |
|  | PREDICT-1 | Beta-cryptoxanthin | 0.0084 | <0.001 | 0.0066 | 0.003 |
|  |  | Carotene diol 1 | 0.0062 | 0.005 | 0.0057 | 0.006 |
|  |  | Carotene diol 2 | 0.0072 | 0.002 | 0.0063 | 0.002 |
|  |  | Carotene diol 3 | 0.0047 | 0.039 | 0.0043 | 0.048 |
| stool | TwinsUK | retinol | 0.0014 | 0.106 | 0.0015 | 0.065 |
|  |  | Beta-cryptoxanthin | 0.0021 | <0.001 | 0.0020 | 0.002 |
|  |  | Carotene diol 1 | 0.0014 | 0.054 | 0.0013 | 0.149 |
|  |  | Carotene diol 2 | 0.0012 | 0.168 | 0.0012 | 0.169 |
|  |  | Carotene diol 3 | 0.0017 | 0.018 | 0.0018 | 0.006 |
|  | PREDICT-1 | Beta-cryptoxanthin | 0.0089 | <0.001 | 0.0085 | 0.001 |
|  |  | Carotene diol 1 | 0.0039 | 0.131 | 0.0034 | 0.275 |
|  |  | Carotene diol 2 | 0.0052 | 0.012 | 0.0050 | 0.016 |
|  |  | Carotene diol 3 | 0.0058 | 0.005 | 0.0058 | 0.005 |

# **Table S4.** Association between circulating vitamin A related metabolites and bacterial species adjusting for age, sex, BMI, family relatedness and multiple testing in TwinsUK

| Vitamin A metabolite | Family | Species | Beta[95%CI] | P | Bonferroni |
| --- | --- | --- | --- | --- | --- |
| Beta-cryptoxanthin | Lachnospiraceae | Blautia_producta | -0.21 [-0.29; -0.13] | 8.73E-07 | 1.61E-03 |
| Beta-cryptoxanthin | Lachnospiraceae | GGB3433_SGB4573 | -0.21 [-0.29; -0.14] | 4.41E-08 | 8.12E-05 |
| Beta-cryptoxanthin | Lachnospiraceae | Lachnospira_eligens | 0.17 [0.11; 0.23] | 1.66E-08 | 3.07E-05 |
| Beta-cryptoxanthin | Lachnospiraceae | Mediterraneibacter_glycyrrhizinilyticus | -0.18 [-0.25; -0.11] | 1.80E-06 | 3.32E-03 |
| Beta-cryptoxanthin | Lachnospiraceae | Ruminococcus_gnavus | -0.15 [-0.21; -0.08] | 2.69E-05 | 4.95E-02 |
| Beta-cryptoxanthin | Pasteurellaceae | Haemophilus_parainfluenzae | 0.16 [0.09; 0.23] | 1.37E-05 | 2.52E-02 |
| Beta-cryptoxanthin | Ruminococcaceae | GGB9615_SGB15053 | 0.14 [0.07; 0.2] | 1.7E-05 | 3.14E-02 |
| Beta-cryptoxanthin | Ruminococcaceae | GGB9707_SGB15229 | 0.14 [0.08; 0.21] | 8.95E-06 | 1.65E-02 |
| Beta-cryptoxanthin | Ruminococcaceae | Ruminococcaceae_unclassified_SGB15233 | 0.16 [0.09; 0.22] | 3.81E-06 | 7.01E-03 |
| Beta-cryptoxanthin | Ruminococcaceae | Ruminococcaceae_unclassified_SGB15236 | 0.17 [0.1; 0.23] | 2.84E-07 | 5.23E-04 |
| Carotene diol (1) | Bacilli_unclassified | Bacilli_bacterium | 0.15 [0.09; 0.21] | 2.95E-06 | 5.43E-03 |
| Carotene diol (1) | Clostridiaceae | Clostridium_sp_AF20_17LB | 0.15 [0.09; 0.22] | 4.12E-06 | 7.59E-03 |
| Carotene diol (1) | Eggerthellaceae | Eggerthella_lenta | -0.12 [-0.17; -0.06] | 1.3E-05 | 2.39E-02 |
| Carotene diol (1) | Lachnospiraceae | Coprococcus_eutactus | 0.13 [0.07; 0.18] | 6.10E-06 | 1.12E-02 |
| Carotene diol (1) | Lachnospiraceae | GGB3746_SGB5089 | 0.12 [0.07; 0.18] | 1.86E-05 | 3.42E-02 |
| Carotene diol (1) | Lachnospiraceae | Lachnospira_eligens | 0.15 [0.1; 0.21] | 3.56E-08 | 6.56E-05 |
| Carotene diol (1) | Lachnospiraceae | Lachnospiraceae_unclassified_SGB4894 | 0.15 [0.08; 0.22] | 8.89E-06 | 1.64E-02 |
| Carotene diol (1) | Lachnospiraceae | Mediterraneibacter_glycyrrhizinilyticus | -0.17 [-0.23; -0.1] | 8.35E-07 | 1.54E-03 |
| Carotene diol (1) | Lachnospiraceae | Ruminococcus_torques | -0.11 [-0.16; -0.06] | 2.31E-05 | 4.25E-02 |
| Carotene diol (1) | Pasteurellaceae | Haemophilus_parainfluenzae | 0.18 [0.12; 0.24] | 3.88E-08 | 7.15E-05 |
| Carotene diol (1) | Ruminococcaceae | Faecalibacterium_SGB15346 | 0.14 [0.08; 0.19] | 7.85E-07 | 1.45E-03 |
| Carotene diol (1) | Ruminococcaceae | GGB9615_SGB15051 | 0.16 [0.09; 0.23] | 4.62E-06 | 8.52E-03 |
| Carotene diol (1) | Ruminococcaceae | GGB9615_SGB15053 | 0.14 [0.09; 0.2] | 6.33E-07 | 1.17E-03 |
| Carotene diol (1) | Ruminococcaceae | GGB9618_SGB15065 | 0.17 [0.09; 0.24] | 6.69E-06 | 1.23E-02 |
| Carotene diol (1) | Ruminococcaceae | GGB9677_SGB15180 | 0.15 [0.09; 0.22] | 3.26E-06 | 6.00E-03 |
| Carotene diol (1) | Ruminococcaceae | GGB9707_SGB15229 | 0.13 [0.08; 0.19] | 5.23E-06 | 9.64E-03 |
| Carotene diol (1) | Ruminococcaceae | Ruminococcaceae_unclassified_SGB15233 | 0.13 [0.07; 0.19] | 1.35E-05 | 2.49E-02 |
| Carotene diol (1) | Ruminococcaceae | Ruminococcaceae_unclassified_SGB15236 | 0.13 [0.08; 0.19] | 4.22E-06 | 7.78E-03 |
| Carotene diol (1) | Ruminococcaceae | Ruminococcaceae_unclassified_SGB4191 | 0.15 [0.09; 0.21] | 6.95E-07 | 1.28E-03 |
| Carotene diol (2) | Bacilli_unclassified | Bacilli_bacterium | 0.16 [0.1; 0.23] | 1.02E-06 | 1.87E-03 |
| Carotene diol (2) | Clostridia_unclassified | GGB4585_SGB6340 | 0.15 [0.08; 0.21] | 1.81E-05 | 3.34E-02 |
| Carotene diol (2) | Clostridia_unclassified | GGB9758_SGB15368 | 0.16 [0.1; 0.21] | 2.28E-07 | 4.19E-04 |
| Carotene diol (2) | Clostridiaceae | Clostridium_sp_AF20_17LB | 0.17 [0.1; 0.24] | 3.91E-07 | 7.21E-04 |
| Carotene diol (2) | Clostridiaceae | Clostridium_sp_AF36_4 | 0.13 [0.07; 0.19] | 8.89E-06 | 1.64E-02 |
| Carotene diol (2) | Clostridiaceae | Clostridium_sp_AM33_3 | 0.13 [0.07; 0.2] | 2.39E-05 | 4.39E-02 |
| Carotene diol (2) | Lachnospiraceae | Blautia_caecimuris | -0.15 [-0.22; -0.08] | 2.18E-05 | 4.02E-02 |
| Carotene diol (2) | Lachnospiraceae | Blautia_glucerasea | 0.14 [0.08; 0.2] | 3.03E-06 | 5.58E-03 |
| Carotene diol (2) | Lachnospiraceae | Coprococcus_eutactus | 0.14 [0.08; 0.2] | 1.02E-06 | 1.88E-03 |
| Carotene diol (2) | Lachnospiraceae | Enterocloster_bolteae | -0.14 [-0.21; -0.08] | 1.69E-05 | 3.11E-02 |
| Carotene diol (2) | Lachnospiraceae | GGB3571_SGB4778 | 0.12 [0.07; 0.18] | 1.36E-05 | 2.51E-02 |
| Carotene diol (2) | Lachnospiraceae | Lachnospira_eligens | 0.17 [0.11; 0.22] | 3.49E-09 | 6.42E-06 |
| Carotene diol (2) | Lachnospiraceae | Lachnospiraceae_bacterium | 0.11 [0.06; 0.16] | 2E-05 | 3.68E-02 |
| Carotene diol (2) | Lachnospiraceae | Lachnospiraceae_bacterium_BX3 | 0.16 [0.09; 0.24] | 1.14E-05 | 2.10E-02 |
| Carotene diol (2) | Lachnospiraceae | Lachnospiraceae_bacterium_OF09_6 | 0.16 [0.09; 0.22] | 2.73E-06 | 5.02E-03 |
| Carotene diol (2) | Lachnospiraceae | Lachnospiraceae_unclassified_SGB4882 | 0.17 [0.1; 0.23] | 1.30E-06 | 2.39E-03 |
| Carotene diol (2) | Lachnospiraceae | Lachnospiraceae_unclassified_SGB4894 | 0.19 [0.12; 0.26] | 2.93E-08 | 5.39E-05 |
| Carotene diol (2) | Lachnospiraceae | Mediterraneibacter_glycyrrhizinilyticus | -0.19 [-0.26; -0.13] | 1.70E-08 | 3.13E-05 |
| Carotene diol (2) | Lachnospiraceae | Ruminococcus_gnavus | -0.14 [-0.2; -0.08] | 1.1E-05 | 2.03E-02 |
| Carotene diol (2) | Lachnospiraceae | Ruminococcus_torques | -0.11 [-0.17; -0.06] | 2.29E-05 | 4.23E-02 |
| Carotene diol (2) | Oscillospiraceae | Oscillibacter_sp_ER4 | 0.12 [0.07; 0.18] | 1.59E-05 | 2.92E-02 |
| Carotene diol (2) | Pasteurellaceae | Haemophilus_parainfluenzae | 0.21 [0.15; 0.28] | 2.33E-10 | 4.29E-07 |
| Carotene diol (2) | Ruminococcaceae | Faecalibacterium_prausnitzii | 0.12 [0.07; 0.18] | 5.15E-06 | 9.49E-03 |
| Carotene diol (2) | Ruminococcaceae | Faecalibacterium_SGB15346 | 0.16 [0.11; 0.22] | 1.04E-08 | 1.91E-05 |
| Carotene diol (2) | Ruminococcaceae | GGB9615_SGB15051 | 0.19 [0.12; 0.26] | 1.56E-07 | 2.86E-04 |
| Carotene diol (2) | Ruminococcaceae | GGB9615_SGB15053 | 0.18 [0.12; 0.23] | 1.96E-09 | 3.62E-06 |
| Carotene diol (2) | Ruminococcaceae | GGB9618_SGB15065 | 0.19 [0.12; 0.27] | 4.54E-07 | 8.36E-04 |
| Carotene diol (2) | Ruminococcaceae | GGB9677_SGB15180 | 0.16 [0.1; 0.23] | 8.86E-07 | 1.63E-03 |
| Carotene diol (2) | Ruminococcaceae | GGB9705_SGB15225 | 0.15 [0.09; 0.21] | 4.73E-07 | 8.71E-04 |
| Carotene diol (2) | Ruminococcaceae | GGB9707_SGB15229 | 0.19 [0.13; 0.25] | 1.30E-10 | 2.39E-07 |
| Carotene diol (2) | Ruminococcaceae | Ruminococcaceae_unclassified_SGB15233 | 0.17 [0.11; 0.23] | 9.98E-09 | 1.84E-05 |
| Carotene diol (2) | Ruminococcaceae | Ruminococcaceae_unclassified_SGB15236 | 0.18 [0.12; 0.23] | 2.05E-09 | 3.78E-06 |
| Carotene diol (2) | Ruminococcaceae | Ruminococcaceae_unclassified_SGB4191 | 0.15 [0.09; 0.21] | 2.62E-06 | 4.82E-03 |
| Carotene diol (3) | Clostridiaceae | Clostridium_sp_AF20_17LB | 0.18 [0.1; 0.25] | 2.07E-06 | 3.82E-03 |
| Carotene diol (3) | Lachnospiraceae | Coprococcus_eutactus | 0.14 [0.08; 0.21] | 1.29E-05 | 2.38E-02 |
| Carotene diol (3) | Lachnospiraceae | Lachnospira_eligens | 0.15 [0.08; 0.21] | 3.84E-06 | 7.07E-03 |
| Carotene diol (3) | Lachnospiraceae | Lachnospiraceae_bacterium | 0.13 [0.07; 0.18] | 1.95E-05 | 3.59E-02 |
| Carotene diol (3) | Lachnospiraceae | Lachnospiraceae_unclassified_SGB4894 | 0.17 [0.1; 0.25] | 4.66E-06 | 8.58E-03 |
| Carotene diol (3) | Lachnospiraceae | Mediterraneibacter_glycyrrhizinilyticus | -0.17 [-0.25; -0.1] | 8.42E-06 | 1.55E-02 |
| Carotene diol (3) | Ruminococcaceae | Faecalibacterium_SGB15346 | 0.15 [0.09; 0.21] | 2.59E-06 | 4.77E-03 |
| Carotene diol (3) | Ruminococcaceae | GGB9615_SGB15051 | 0.17 [0.09; 0.25] | 1.37E-05 | 2.53E-02 |
| Carotene diol (3) | Ruminococcaceae | GGB9615_SGB15053 | 0.17 [0.11; 0.24] | 5.82E-08 | 1.07E-04 |
| Carotene diol (3) | Ruminococcaceae | GGB9707_SGB15229 | 0.17 [0.11; 0.24] | 1.76E-07 | 3.24E-04 |
| Carotene diol (3) | Ruminococcaceae | Ruminococcaceae_unclassified_SGB15236 | 0.16 [0.09; 0.22] | 1.91E-06 | 3.53E-03 |
| Carotene diol (3) | Ruminococcaceae | Ruminococcaceae_unclassified_SGB4191 | 0.16 [0.1; 0.23] | 2.68E-06 | 4.94E-03 |
| Oxoretinoic acid | Bacilli_unclassified | Bacilli_bacterium | 0.16 [0.09; 0.23] | 1.22E-05 | 2.25E-02 |
| Oxoretinoic acid | Barnesiellaceae | Barnesiella_intestinihominis | 0.13 [0.07; 0.19] | 1.13E-05 | 2.08E-02 |
| Oxoretinoic acid | Clostridia_unclassified | Clostridia_bacterium | 0.14 [0.08; 0.19] | 7.81E-06 | 1.44E-02 |
| Oxoretinoic acid | Clostridia_unclassified | GGB9758_SGB15368 | 0.15 [0.08; 0.21] | 7.64E-06 | 1.41E-02 |
| Oxoretinoic acid | Clostridia_unclassified | GGB9787_SGB15410 | 0.16 [0.09; 0.23] | 2.15E-06 | 3.96E-03 |
| Oxoretinoic acid | Eggerthellaceae | GGB45432_SGB63101 | 0.14 [0.08; 0.2] | 5.83E-06 | 1.07E-02 |
| Oxoretinoic acid | Lachnospiraceae | Blautia_caecimuris | -0.2 [-0.28; -0.13] | 3.52E-07 | 6.49E-04 |
| Oxoretinoic acid | Lachnospiraceae | Blautia_producta | -0.19 [-0.27; -0.11] | 6.91E-06 | 1.27E-02 |
| Oxoretinoic acid | Lachnospiraceae | Blautia_SGB4815 | 0.15 [0.09; 0.2] | 1.05E-06 | 1.93E-03 |
| Oxoretinoic acid | Lachnospiraceae | Blautia_SGB4831 | 0.16 [0.1; 0.22] | 4.88E-07 | 8.99E-04 |
| Oxoretinoic acid | Lachnospiraceae | Coprococcus_eutactus | 0.14 [0.07; 0.2] | 2.12E-05 | 3.91E-02 |
| Oxoretinoic acid | Lachnospiraceae | Dorea_sp_AF24_7LB | 0.13 [0.07; 0.2] | 1.62E-05 | 2.99E-02 |
| Oxoretinoic acid | Lachnospiraceae | GGB3571_SGB4778 | 0.16 [0.1; 0.22] | 4.53E-07 | 8.35E-04 |
| Oxoretinoic acid | Lachnospiraceae | Lachnospira_eligens | 0.13 [0.07; 0.2] | 1.3E-05 | 2.40E-02 |
| Oxoretinoic acid | Lachnospiraceae | Mediterraneibacter_glycyrrhizinilyticus | -0.22 [-0.29; -0.14] | 5.63E-09 | 1.04E-05 |
| Oxoretinoic acid | Peptostreptococcaceae | Intestinibacter_SGB6139 | 0.15 [0.08; 0.22] | 2.41E-05 | 4.45E-02 |
| Oxoretinoic acid | Rikenellaceae | Alistipes_shahii | 0.15 [0.09; 0.21] | 1.65E-06 | 3.04E-03 |
| Oxoretinoic acid | Ruminococcaceae | GGB9602_SGB15031 | 0.15 [0.08; 0.21] | 8.60E-06 | 1.58E-02 |
| Oxoretinoic acid | Ruminococcaceae | GGB9635_SGB15106 | 0.15 [0.08; 0.21] | 0.000011 | 2.03E-02 |
| Oxoretinoic acid | Ruminococcaceae | GGB9707_SGB15229 | 0.16 [0.09; 0.22] | 1.28E-06 | 2.36E-03 |
| Oxoretinoic acid | Ruminococcaceae | Ruminococcaceae_unclassified_SGB15234 | 0.18 [0.12; 0.25] | 2.75E-08 | 5.07E-05 |
| Oxoretinoic acid | Ruminococcaceae | Ruminococcaceae_unclassified_SGB15236 | 0.15 [0.09; 0.21] | 4.37E-06 | 8.05E-03 |

# **Table S5.** Association between Shannon and HEI, adjusted for age, sex, BMI, and family relatedness and the below carotenoids.

|  | Beta | SE | p |
| --- | --- | --- | --- |
| Covariates only | 0.10 | 0.04 | 0.01 |
| Carotenediol1 | 0.07 | 0.04 | 0.08 |
| Carotenediol2 | 0.07 | 0.04 | 0.07 |
| Carotenediol3 | 0.08 | 0.04 | 0.05 |
| Beta-cryptoxanthin | 0.08 | 0.04 | 0.05 |

# **Table S6.** Sensitivity Analysis for Unmeasured Confounding using the E-value method.

|  | E-value | Lower bound CI |
| --- | --- | --- |
| Carotenediol1 | 1.32 | 1 |
| Carotenediol2 | 1.32 | 1 |
| Carotenediol3 | 1.35 | 1.04 |
| Beta-cryptoxanthin | 1.35 | 1.04 |

**Figure S1.** Association between vitamin A related metabolites and circulating primary bile acids. Labels represent Beta coefficients and p values in parenthesis. Non-significant correlations are semi-transparent.


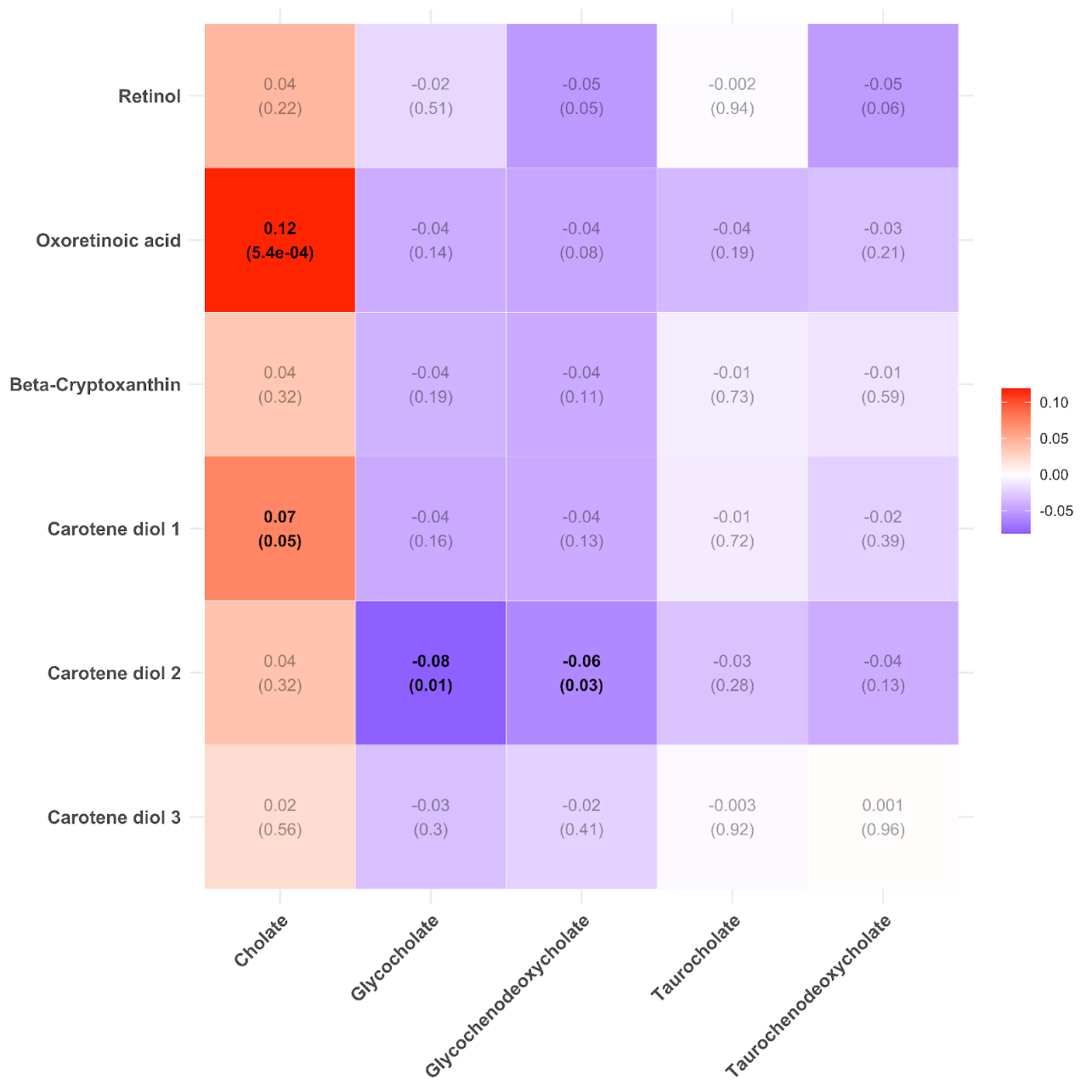

Supplement: Supplementary file 1 — Supplementary Material 1: Table S1. Association between Shannon Diversity and vitamin A related metabolites adjusting for age, sex, BMI, history of cardiovascular diseases (i.e. cerebrovascular disease, heart failure, ischemic heart disease, coronary artery disease and atrial fibrillation), type 2 diabetes, chronic obstructive pulmonary disease, allergy, diet (i.e. healthy eating index, fibre intake, vegetable intake and energy intake), antibiotics use, physical exercise, vitamin supplementation, sequencing depth and family relatedness in TwinsUK. In this analysis, missing dietary values were imputed to the mean. Table S2. Association between Alpha Diversity indices and vitamin A related metabolites adjusting for age, sex, BMI and family relatedness in TwinsUK and PREDICT-1. Beta (SE) and P value for fixed effect meta-analysis are reported. Table S3. Beta-diversity estimates for gut microbiome composition in relation to serum and stool vitamin A-related metabolites using PERMANOVA (Bray-Curtis dissimilarity). Results of the PERMANOVA analysis as implemented in ADONIS, examining the association between gut microbiome beta-diversity using Bray-Curtis dissimilarity and serum and stool vitamin A-related metabolites. The table includes the R-squared (R²) values, and p-values for each metabolite unadjusted and adjusted for covariates (age, sex, BMI and family relatedness). The R² value represents the proportion of total variation in gut microbiome composition explained by each metabolite. P-values are derived from 1000 permutations and indicate the statistical significance of the observed associations. Table S4. Association between circulating vitamin A-related metabolites and bacterial species adjusting for age, sex, BMI, family relatedness and multiple testing in TwinsUK. Table S5. Association between Shannon and HEI, adjusted for age, sex, BMI, and family relatedness and the below carotenoids. Table S6. Sensitivity Analysis for Unmeasured Confounding using the E-value [file 12916_2024_3543_MOESM1_ESM.docx]
